# Butyric acid ameliorates PCOS-related reproductive dysfunction through gut-brain-ovary axis signaling and ovarian steroidogenic factor activation

**DOI:** 10.3389/fendo.2025.1604302

**Published:** 2025-07-09

**Authors:** Xueping Feng, Juan Xiao, Decai Wang, Xianzhao Fu, Jie Gao, Minli Jiang, Jin Li, Lihe Jiang, Xingwei Liang, Yanna Huang, Qinyang Jiang

**Affiliations:** ^1^ School of Basic Medicine, Youjiang Medical University for Nationalities, Baise, China; ^2^ College of Animal Science and Technology, Guangxi University, Nanning, China; ^3^ Guangxi Key Laboratory of Molecular Medicine in Liver Injury and Repair, The Affiliated Hospital of Guilin Medical University, Guilin, China; ^4^ Traditional Chinese Medicine department, The Affiliated Hospital of Youjiang Medical University for Nationalities, Baise, China

**Keywords:** sodium butyrate, polycystic ovary syndrome, reproductive performance, gut-brain-ovary axis, steroidogenic factor

## Abstract

**Background:**

Butyric acid deficiency is implicated in polycystic ovary syndrome (PCOS), as evidenced by reduced levels in both clinical and preclinical models. Sodium butyrate (NaBu),a butyric acid substitute, has demonstrated therapeutic potential through gut-brain axis modulation, anti-inflammatory effects, and reproductive function protection. This study investigates NaBu’s mechanistic role in PCOS pathophysiology.

**Methods:**

PCOS rats received lipo-coated NaBu diet for three weeks. Systemic and tissue analyses included: serum hormone profiling, lipid metabolism assessment, ovarian/colonic histopathology, Short-chain fatty acids (SCFAs) analysis, and proteomics analysis. Primary granulosa cell cultures with lentiviral transfection elucidated molecular mechanisms. Reproductive performance was evaluated longitudinally.

**Results:**

Treatment with NaBu in PCOS rats resulted in reduced food intake, inhibited weight gain, improved abnormal lipid metabolism, restored estrus cycles and ovulation, lower serum levels of testosterone (T), insulin (INS), and luteinizing hormone (LH), and higher levels of estradiol (E_2_) and progesterone (P_4_). Additionally, NaBu treatment improved the morphology of polycystic ovaries, elevated colonic levels of G protein-coupled receptor 41 (GPR41), peptide tyrosine-tyrosine (PYY), and butyric acid, and enhanced reproductive performance in PCOS rats. Proteomic analysis and cell experiments suggested that upregulation of Cytochrome P450 1b1 (Cyp1b1) may play a crucial role in regulating E_2_ metabolism and P_4_ production, potentially contributing to the pathogenesis of PCOS and ovarian dysfunction.

**Conclusion:**

These findings indicate that NaBu may exert its regulatory effects on appetite and hormone levels in the hypothalamus through the gut-brain-ovary axis, modulating the expression of ovarian steroidogenic factors, thereby improving follicular development and granulosa cell function, and enhancing reproductive performance.

## 1 Introduction

Polycystic ovary syndrome (PCOS) is a common endocrine-metabolic disease in women, with an incidence of up to 5% ~20%, its main characteristics include hyperandrogenemia, anovulation/oligovulation and polycystic ovarian morphology (1). Currently, clinical treatments for patients with PCOS mainly involve three categories: medication, non-pharmacological interventions, and assisted reproductive technologies. While these approaches can alleviate PCOS symptoms to varying extents, they inevitably bring about certain side effects or limitations (2). Effectively and safely treating PCOS remains a challenge in reproductive medicine.

A considerable number of studies have shown that the occurrence of PCOS is closely related to the dysbiosis of gut microbiota (3–5). The gut microbiota closely connects external signals and the immune system, where SCFAs act as essential mediators, modulating immune responses, or maintaining metabolic homeostasis in the host (6). Acetic acid, propionic acid and butyric acid are the most important SCFAs. Among them, Butyric acid has received the most attention. Research found that colonic fecal butyric acid levels were lower in PCOS rats (7, 8). Interestingly, low levels of butyric acid were also detected in the fecal of PCOS patients (9, 10), which suggests that reduced levels of butyric acid are associated with PCOS. NaBu, one of the butyrate, is often used in place of butyric acid in animal studies and practical industry applications due to its physical properties, including being solid, stable, and much less odorous (11, 12). NaBu has been reported to mitigate the development of metabolic syndrome through its anti-inflammatory, anti-oxidative, enhanced insulin sensitivity, lipid-lowering effects and amelioration of hepatic steatosis (13–16).

NaBu has drawn particular attention due to its beneficial effects on intestinal and brain functions, such as colonic homeostasis and blood-brain barrier permeability (17–19). Research indicates that NaBu can exert an influence on the brain through the gut-brain axis, regulating hormones and inflammation, either as an energy source or by binding to G protein-coupled receptors (20, 21). Additionally, it has protective and beneficial effects on animal reproduction (22–27).The functions and advantages of NaBu prompted us to investigate its potential in alleviating PCOS symptoms. Therefore, this study investigates the impact of NaBu on PCOS rats by feeding them a diet containing lipo-coated NaBu. This approach allows NaBu to reach the colonic region of rats upon ingestion, simulating the pathway of butyric acid production. The results revealed that NaBu treatment significantly mitigated estrous cycle disruption, hormonal imbalances, ovarian morphological abnormalities, abnormal lipid metabolites, and improved reproductive performance in PCOS rats. This work will contribute to deepening our understanding of the pathogenesis of PCOS from a new perspective and indicate that NaBu may be a strategy for treating PCOS.

## 2 Materials and methods

### 2.1 Materials

The following primary antibodies: Anti-Cyp1b1 (Cat#DF6399), Anti-GPR41 (Cat#AF9057) and anti-GAPDH (Cat#AF7021) was purchased from Affinity Biosciences Technology (Jiangsu, China). PrimeScript™ RT reagent Kit with gDNA Eraser (RR047A) and TB Green^®^ Premix Ex Taq™ II (RR820A) were purchased from TaKaRa(Dalian, China)and PCR primers were purchased from Gencreat (Wuhan, China). RIPA tissue/cell lysate was purchased from Solarbio life sciences (Beijing, China). The ELISA kits [PYY (SYP-R0196), Ghrelin (RX301269R) and 4-OHE2(RXJ303175R)] were purchased from Ruixin Biotechnology Co., Ltd (Quanzhou, China). TG (A110-1-1), TC (A111-1-1), LDL-C (A113-1-1) and HDL-C (A112-1-1) were purchased from Nanjing Jiancheng Bioengineering Institute (Nanjing, China). Lentivirus GV492 and GV492-cyp1b1 were purchased from Shanghai Genechem Co.,Ltd. Rodent Maintenance feed (1025) and Maintenance powder feed (1024) were purchased from Guangdong Vital River Laboratory Animal Technology Co., Ltd.

### 2.2 Feed and letrozole solution preparation

The lipo-coated NaBu was provided by King Techina Technology Co., Ltd with a purity of 30%. According to previous studies, dietary supplementation with 1% ~5% NaBu has been shown to exert beneficial effects in animal disease models (13, 28, 29). Based on preliminary experimental results, the present study adopted a final NaBu concentration of 3.6% in the diet. Equivalent to adding 12g of lipid-coated NaBu per 1000g of maintenance feed, the effective dose of NaBu is 360mg. Preparation of the treatment feed for PCOS rats: The maintenance powder feed was uniformly blended with lipid-coated NaBu, then process it into feed pellets through granulation for storage.

A 0.5% carboxymethyl cellulose sodium (CMC) solution was prepared by dissolving 0.5 g CMC in 100 mL of boiled double-distilled water with continuous stirring until complete dissolution. After cooling to room temperature, 1 g of letrozole powder was added and thoroughly mixed to prepare a 1 mg/mL working solution. The solution was aliquoted and stored at -20°C.

### 2.3 Animal study design

Eight-week-old female Sprague Dawley rats were purchased from Changsha Tianqin Biotechnology Co., Ltd. [SCXK (Xiang) 2019-0014, No. 430726210100078487] and housed at the Experimental Animal Center of Youjiang Medical University for Nationalities [SYXK (Gui) 2022-0003] at a density of three rats per cage. The rats were maintained in a controlled environment with a temperature of 22 ± 2°C, relative humidity of 55 ± 5%, and a 12/12 hour light/dark cycle. All experimental procedures were approved by the Animal Welfare and Ethics Committee of Youjiang Medical University for Nationalities (No. 2022031005).

After one week of adaptive feeding, the rats were randomly divided into two groups: the normal control group (NC, n=6) and the model group (Model, n=12).The PCOS modeling method refers to the existing literature report (7). In the first stage, the NC group received oral gavage of 0.5% CMC solution (1 mL/kg/day), while the Model group received oral gavage of letrozole solution (1 mg/kg/day) for 28 consecutive days, with weekly weighing. In the second stage, the model group was further divided into the PCOS group and the NaBu group, each consisting of 6 rats. The rats were individually housed in metabolic cages, with the NC and PCOS groups having ad libitum access to maintenance feed, and the NaBu group having feed containing 3.6% NaBu, administered for 21 consecutive days. On day40, the rats were subjected to vaginal smear examination for 10 consecutive days (about 2 estrus cycles). All rats were weighed once a week, and the average food intake was recorded from day29 to day49.

### 2.4 Staining and observation of vaginal smears in rats and sample collection

The vaginal smear of a rat was stained with HE and observed under an optical microscope(Olympus, Japan). The estrus cycle in rat averages 4~5 days and is generally divided into four stages: proestrus, estrus, metestrus, and diestrus. The stages of estrus are distinguished by identifying different cell types, following methods from our previous research (7). At the end of the experiment, all rats were fasted for 12 hours and anesthetized with isoflurane. Blood samples were collected from the tail artery to measure fasting blood glucose levels. Subsequently, the abdominal cavity was opened, and blood was collected from the abdominal aorta. Serum, ovaries, colon, and fecal samples were collected for further experimental analysis.

### 2.5 Serum sample analysis

Blood samples collected from rats were allowed to stand at 4°C overnight and then centrifuged at 4000 rpm for 10 minutes. The supernatant was collected and stored at -80°C for further use. The radioactive immunoassay for serum T, INS, E_2_, P_4_, FSH, and LH levels was conducted by Beijing North Institute of Biological Technology by using their assay kit (220220). The serum levels of Ghrelin, PYY, TC, TG, HDL-C and LDL-C were determined following the instructions provided in the ELISA assay kit.

### 2.6 SCFAs analysis and iTRAQ proteomics analysis

SCFAs analysis of rat feces and iTRAQ proteomics analysis of rat ovaries were both conducted by Nanning Current Science Biotechnology Co., Ltd (Guangxi, China).

### 2.7 Ovary and colon morphology analysis

The ovarian and colon tissue samples were fixed in 4% paraformaldehyde solution at 4°C over 24h, then embedded in paraffin and cut into 3~4μm thickness for hematoxylin-eosin staining. Analysis was performed using an optical microscope (Olympus, Japan). Follicles with a thinned granulosa cell layer and a thickened theca cell layer were recognized as cystic follicles. The numbers of cystic follicles and corpora lutea were counted, and the results including colon morphology analysis were confirmed by a pathologist.

### 2.8 Immunohistochemical analysis

The rat colon and ovarian tissues were fixed in 4% paraformaldehyde at 4°C for 48 hours, followed by dehydration, embedding in paraffin wax, and sectioning. After dewaxing and hydration, sections were subjected to endogenous peroxidase blocking at room temperature for 10 minutes. Subsequently, they were rinsed with PBS buffer (3 min×3 times) and incubated with a primary antibody (1:200) at 37°C overnight. On the following day, sections were rinsed again with PBS buffer (3 min×3 times), treated with goat anti-rabbit IgG polymer for 20 minutes at 37°C, and washed with PBS buffer (3 min×3 times). DAB was used for color development, followed by incubation with hematoxylin staining solution for 20 seconds. After dehydration with alcohol, sealing with xylene transparent and neutral gum, the staining results were observed under an optical microscope.

### 2.9 Quantitative real-time PCR analysis

RNA was extracted from ovarian tissue by Trizol method, cDNA was synthesized by reverse transcription, and the target gene was amplified by quantitative real-time PCR. The primer sequences are shown in **Supplementary Table S1**. The mRNA expression levels of the target genes were normalized by 2^−ΔΔCT^ with β-actin as the internal reference.

### 2.10 Western blot analysis

Initially, 40 mg of ovarian tissue was homogenized in RIPA tissue lysate, and the resulting supernatant was collected after centrifugation. Protein concentration was determined using the BCA method. The protein sample was then mixed with sample buffer, denatured at 100°C for 5 minutes, and the supernatant was obtained after centrifugation. Next, an 8% SDS-PAGE gel was prepared, and proteins were separated by electrophoresis at 100 V for 100 minutes. The proteins were transferred from the gel to a PVDF membrane at 250 mA for 80 minutes, followed by blocking with 5% BSA for 1 hour. After washing with TBST, a primary antibody (1:1000) was added and incubated overnight at 4°C. The following day, TBST washes were performed, and a secondary antibody (1:5000~10000) was added for 1 hour. After additional TBST washes, ECL developer was applied for visualization.

### 2.11 Cell experiment design

Six 30-day-old SPF female SD rats were subcutaneously injected with 50 IU of pregnant mare serum gonadotropin (PMSG) behind the neck to stimulate follicular development. After 48 hours, the rats were euthanized, and soaked in 75% alcohol for disinfection for 30 minutes. The ovaries were aseptically removed and washed twice in PBS containing 2% antibiotics (penicillin 100 IU/mL, streptomycin 100 μg/mL). The surrounding fat tissue was carefully removed, and the ovaries were placed in DMEM/F12 medium containing 2% antibiotics. Follicles were punctured using a 1 mL sterile syringe to collect granulosa cells. The cell suspension was filtered through a 200-mesh stainless steel cell strainer, centrifuged at 1000 rpm for 5 minutes, and the supernatant was discarded. The cell pellet was resuspended in red blood cell lysis buffer and incubated at 37°C for 10 minutes. The mixture was then centrifuged at 1000 rpm for 5 minutes, and the supernatant was discarded. The cells were resuspended in PBS and centrifuged at 1000 rpm for 3 minutes. The cell pellet was collected and resuspended in complete medium containing 10% fetal bovine serum, and cultured at 37°Cwith 5% CO2 for 48 hours before changing the medium. When the cells reached about 70% confluence, they were identified. Granulosa cells were then infected with either the empty lentiviral vector GV492 or the lentiviral vector GV492-cyp1b1 to determine the optimal infection conditions. Finally, the effects of the lentiviral vectors under optimal infection conditions on granulosa cell function were analyzed.

### 2.12 Rat reproduction experiment

The adaptive feeding, grouping and feeding methods of rats are the same as 2.3. Male rats with normal reproductive function were put into cages for 21 days at the second stage in a ratio of 1:3. In the third stage, male rats were taken out at day49, and female rats were kept for 21 days, during which they were fed maintenance diets.

### 2.13 Statistical analysis

Data are expressed as mean ± standard deviation (SD). SPSS 20.0 and GraphPad Prism 8.0 were used for data analysis and mapping, respectively. Image Pro Plus 6.0 and Image J software were used to measure the optical density and protein band gray value of the immunohistochemical results, respectively. For the data conforming to the homogeneity test of variance, LSD(L) in one-way analysis of variance (ANOVAs) was used for statistical analysis. For the data that did not meet the homogeneity test of variance, Kruskal Wallis rank sum test in the non-parametric test was used for statistical analysis, *p* < 0.05 was considered statistically significant.

## 3 Results

### 3.1 NaBu treatment caused weight loss and reduced food intake in PCOS rats

**Figure 1:**
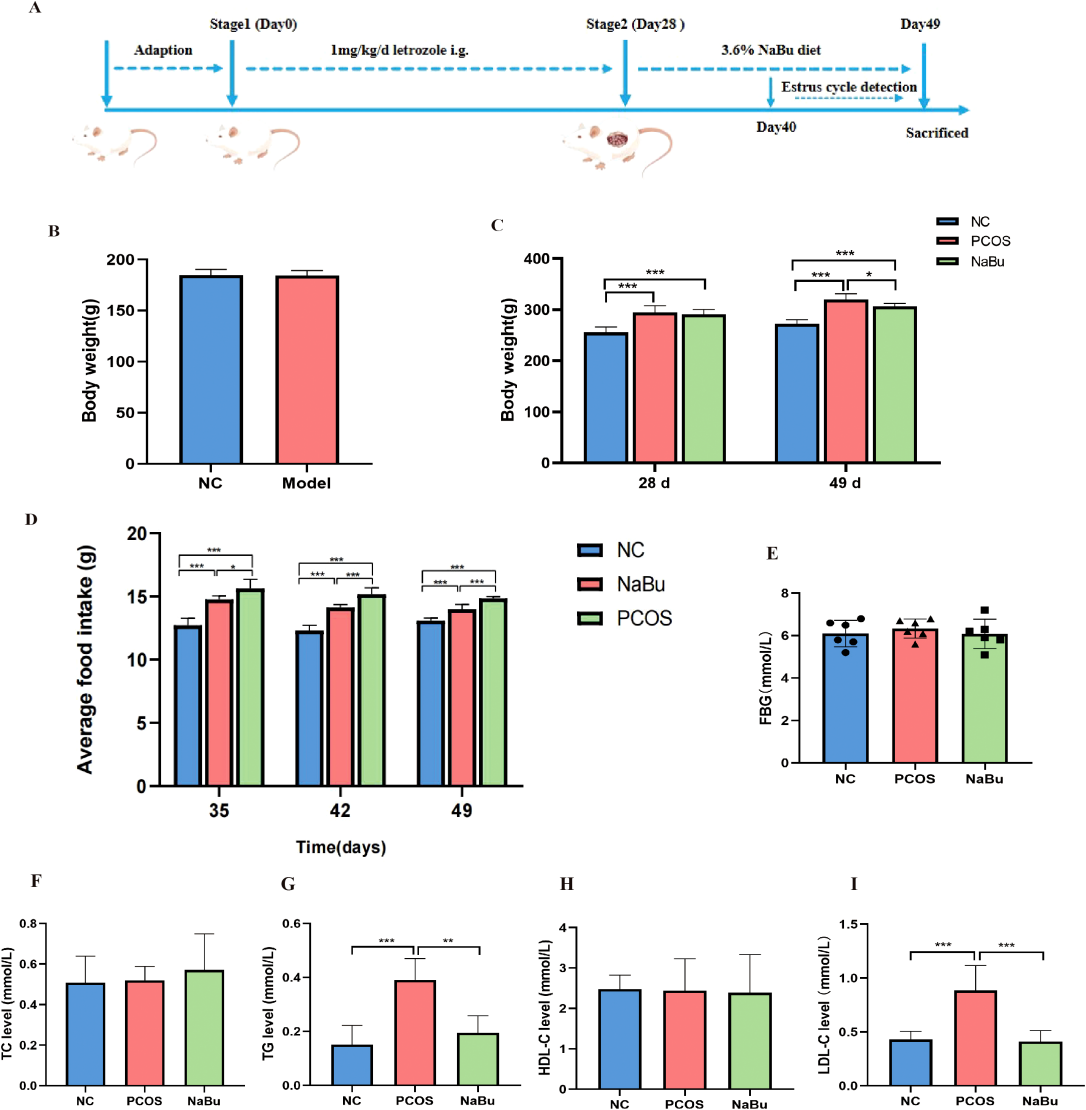
Effects of NaBu treatment on body weight, food intake, FBG and lipid metabolism factors in rats. **(A)** Experimental design process. The whole process included 7 days of adaptation, 28 days of letrozole administration and 21 days of NaBu diet treatment. **(B)**The body weight of the NC group and the Model group before the experiment,n=6/12. **(C)**The body weight before and after NaBu treatment, n=6. **(D)** Average weekly food intake, n=6. **(E)** FBG value detection, n=6. **(F–I)** The levels of TC, TG, HDL-C and LDL-C, n=6. Note: *p <0.05, **p < 0.01, ***p <0.001.

According to the letrozole-induced PCOS rat model and treatment process (**Figure 1A**), although initial body weights did not differ between the NC and model groups (**Figure 1B**), the PCOS and NaBu groups exhibited significantly higher body weights than the NC group after 28 days of letrozole administration. After 21 days of NaBu treatment (day29 to day 49), rats in the NaBu group exhibited significantly lower body weight and reduced average food intake compared to the PCOS group (**Figures 1C, D**). Notably, NaBu treatment showed no significant effect on fasting blood glucose (FBG) levels (**Figure 1E**). Biochemical analysis demonstrated that PCOS rats had significantly elevated triglycerides (TG) and low-density lipoprotein cholesterol (LDL-C) levels relative to NC controls, while total cholesterol (TC) and high-density lipoprotein cholesterol (HDL-C) levels remained comparable between these groups. Importantly, NaBu treatment effectively ameliorated the dyslipidemia in PCOS rats, as evidenced by significant lower in both TG and LDL-C levels (**Figures 1F–I**).

### 3.2 NaBu treatment could restore the estrus cycle in PCOS rats

**Figure 2:**
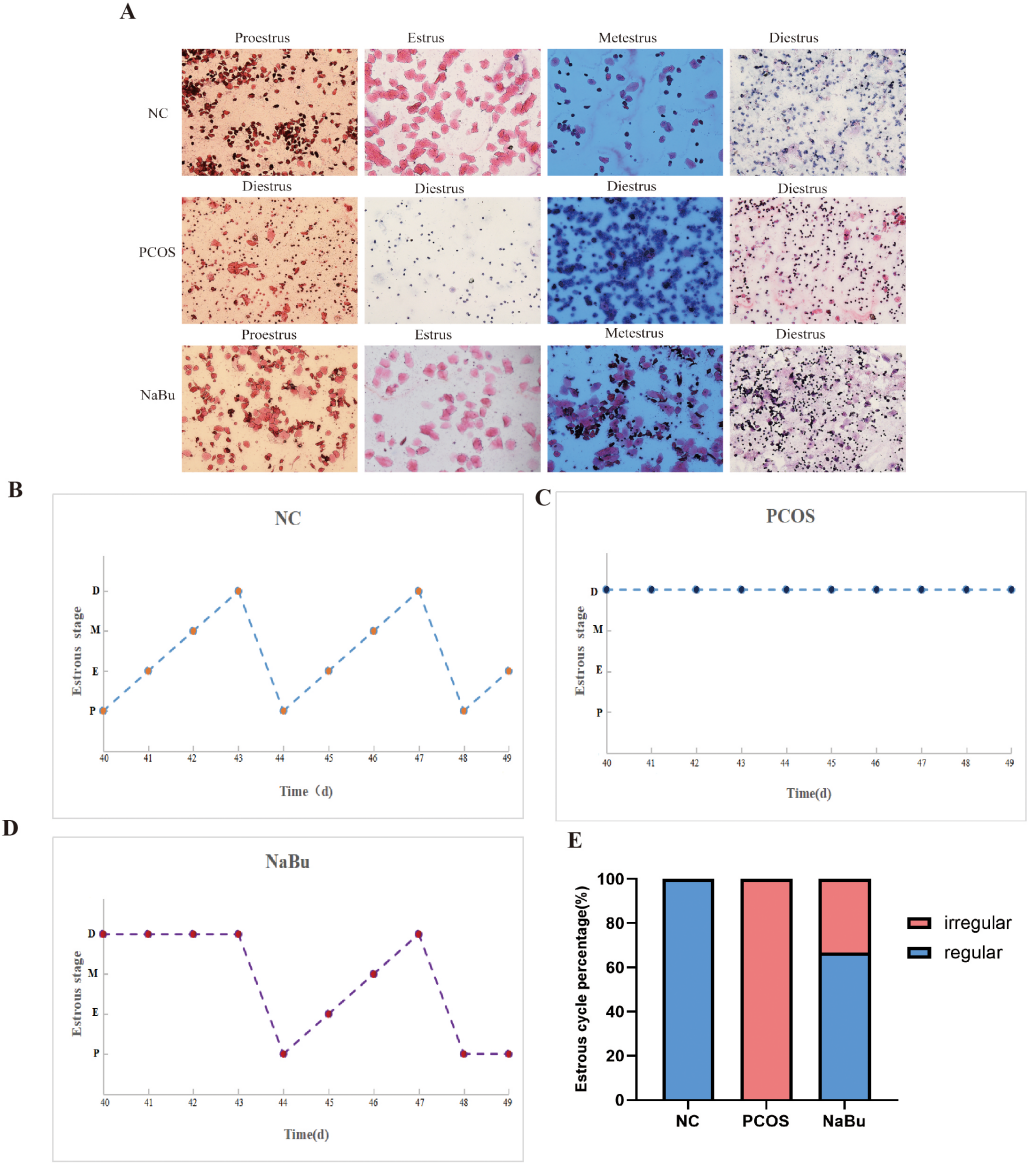
Effects of NaBu treatment on estrus cycle in rats. **(A)** Observation of vaginal cell morphology in rats. D: diestrus, M: metestrus, E: estrus, P: proestrus. **(B–D)** Estrus stage of rats were examined for 10 consecutive days, with only one representative from each group selected for demonstration. **(E)** The proportions of regular and irregular estrus cycles in each group of rats, n=6.

Vaginal smears were performed on the rats for 10 consecutive days from day40 to day49, and the morphology of vaginal exfoliated epithelial cells of rats was observed to distinguish different stages of estrus in order to evaluate the effect of NaBu treatment (**Figure 2A**). The results showed that the rats in the NC group still maintained regular estrus cycles (**Figure 2B**), the rats in the PCOS group were always in the diestrus stage (**Figure 2C**), and the rats in the NaBu group began to show estrus on the day45 (**Figure 2D**). A normal estrus cycle should have four consecutive estrus stages; thus, we counted the estrus stages for each rat. A complete estrus cycle observed is defined as estrus regular and conversely as irregular. According to statistics, all rats in the NC group had regular estrus cycles, while those in the PCOS group were always irregular; 4 out of 6 rats in the NaBu group were observed to have regular estrus cycles, with a recovery rate of 66.7% (**Figure 2E**).

### 3.3 NaBu treatment improved endocrine dysregulation and ovarian polycystic morphology in PCOS rats

**Figure 3:**
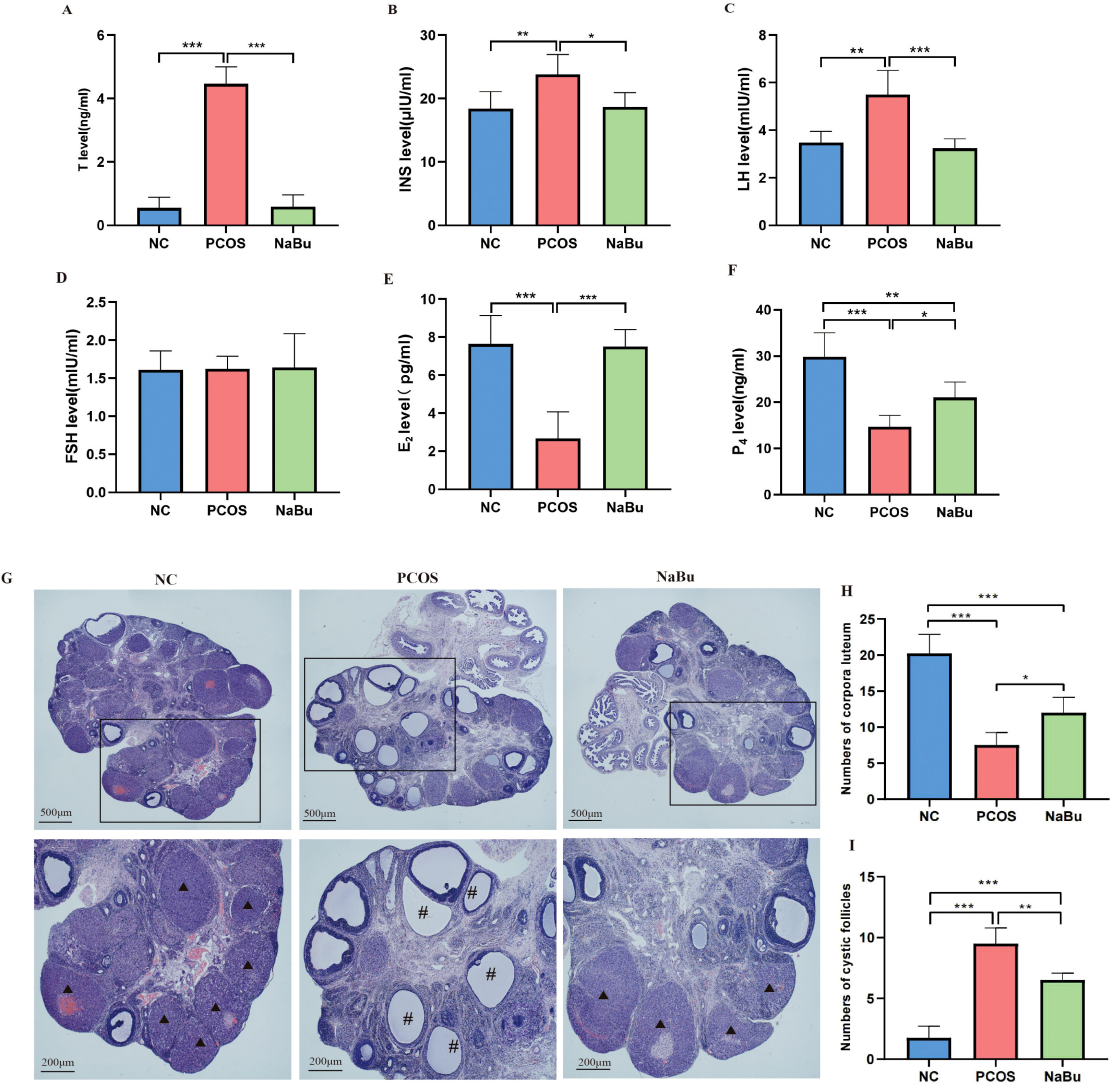
The effects of NaBu treatment on hormone levels and ovarian pathological morphology in rats. **(A–F)**The levels of T, INS, LH, FSH, E_2_ and P_4_ in rat serum, n=6. **(G)** Pathological morphology of ovary in rats. **(H)** Numbers of corpora luteum, n=4. **(I)** Numbers of cystic follicles, n=4. *p <0.05, **p < 0.01, ***p <0.001; ▴ indicates the corpus luteum, # indicates the cystic follicle, scale bar=500 μm/200 μm.

To evaluate the effects of NaBu on endocrine hormones in rats, serum was collected for hormonal assays. Compared with the NC group, the PCOS group showed significantly higher levels of T, LH, and INS (**Figures 3A–C**) but lower levels of E2 and P4 (**Figures 3E, F**), while FSH was not significantly different (**Figure 3D**). After NaBu treatment, T, LH and INS were significantly lower while E_2_ and P_4_ were significantly higher, suggesting that NaBu treatment could reverse endocrine disorders in PCOS rats.

The ovaries of NC group rats displayed normal histological architecture, featuring multiple well-developed corpora lutea and follicles at various developmental stages. In stark contrast, the PCOS model group exhibited characteristic pathological changes, including a marked increase in cystic follicles, significant reduction in corpora lutea count, and notable thinning of the granulosa cell layer. Notably, NaBu treatment substantially attenuated these polycystic ovarian alterations, as evidenced by the restoration of corpora lutea numbers and the reappearance of a well-organized, thickened granulosa cell layer (**Figures 3G–I**).

### 3.4 NaBu treatment elevated fecal propionic acid and butyric acid levels, and was associated with higher GPR41 expression and PYY secretion in the colon of PCOS rats

**Figure 4:**
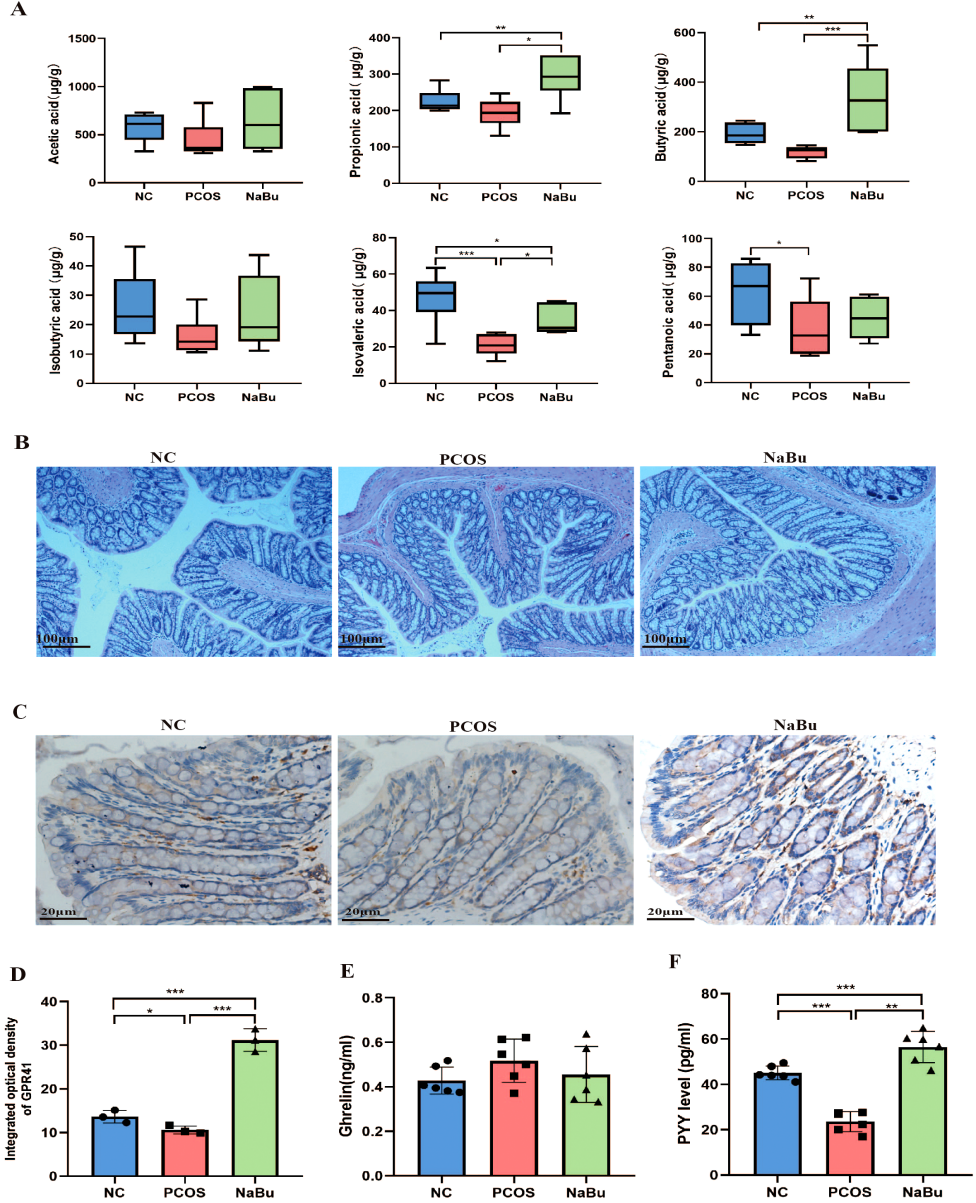
Effects of NaBu treatment on SCFAs, GPR41 and intestinal hormone in rats. **(A)** The levels of SCFAs in rat feces, n=6. **(B)** HE staining of rat colon. **(C)** Immunohistochemical staining of GPR41 factor in rat colon. **(D)** GPR41 integrated optical density, n=3. **(E, F)** The levels of Ghrelin and PYY in rat serum, n=6. Note: scale bar=100 μm/20 μm; *p <0.05, **p < 0.01, ***p <0.001.

SCFAs profiles (**Figure 4A**) revealed that compared with NC group, the levels of propionic acid and butyric acid in NaBu group were significantly higher, while isovaleric acid was significantly lower; the levels of pentanoic acid and isovaleric acid in PCOS group lower significantly. Compared with PCOS group, the levels of propionic acid, butyric acid and isovaleric acid in NaBu group were significantly higher. There were no significant differences in acetic acid levels between the groups.

Histopathological examination of rat colonic tissues revealed that NaBu treatment did not significantly alter colonic morphology. The mucosal architecture remained intact in all experimental groups, demonstrating that NaBu treatment induces no adverse effects on intestinal histology (**Figure 4B**). Immunohistochemical analysis showed that the expression level of colon GPR41 protein was significantly up-regulated in the NaBu group (**Figures 4C, D**). Serum PYY levels in PCOS rats were significantly higher after NaBu intervention, but Ghrelin levels were not affected (**Figures 4E, F**). Our findings indicate that NaBu treatment upregulates GPR41 expression in the colon and increases serum PYY levels, suggesting a potential role of GPR41 in mediating PYY secretion.

### 3.5 Proteomic analysis of rat ovary

**Figure 5:**
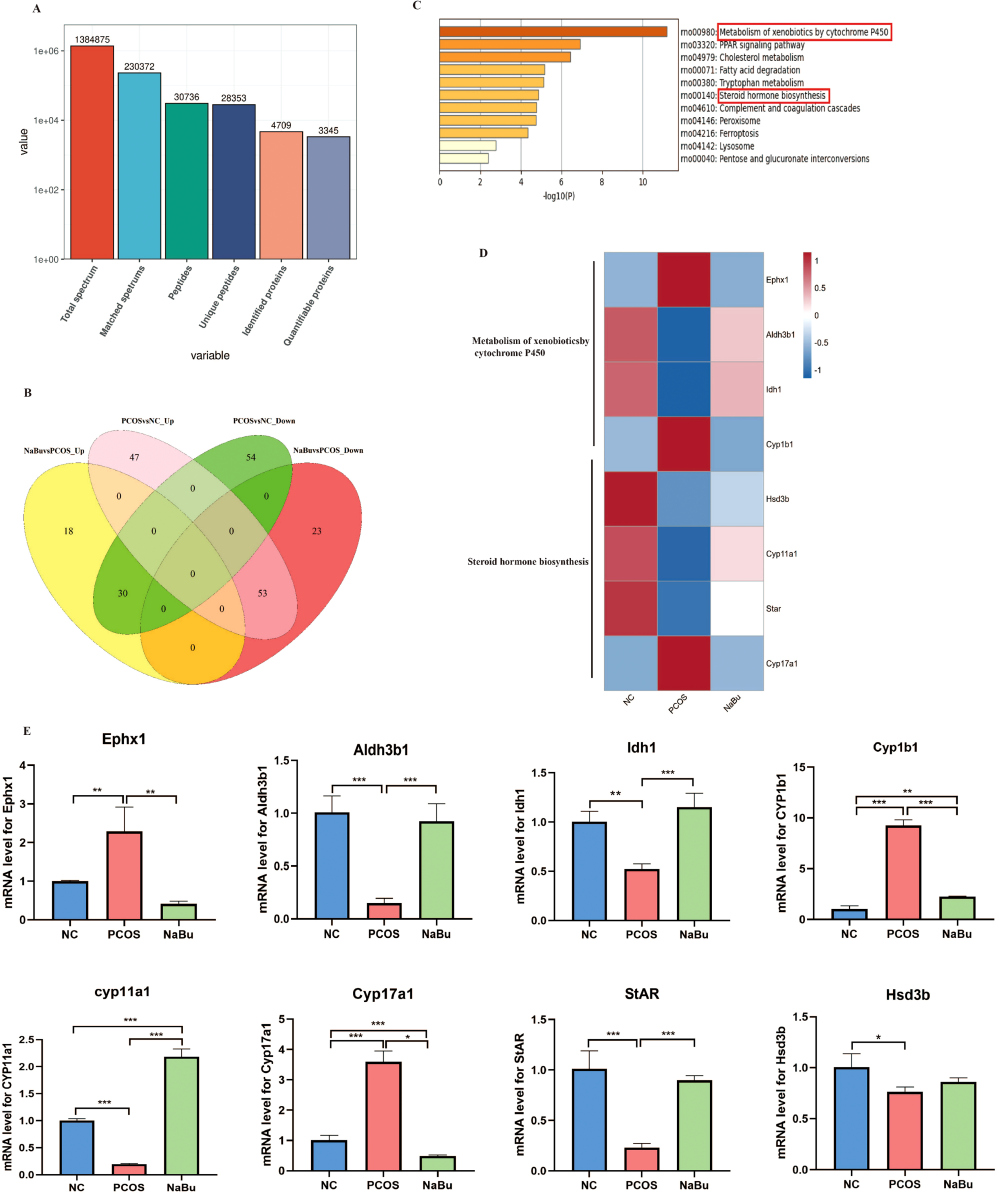
Proteomic analysis of rat ovaries. **(A)** Statistics of protein mass and Oreochronisnilotcus Uniprot database match results. **(B)** Venn diagram showing upregulated and downregulated proteins in PCOS group vs. NC group and in NaBu group vs. PCOS group. **(C)** KEGG enrichment analysis of overlapping DEPs. **(D, E)** Heatmap showing the expression patterns of the overlapping DEPs and the confirmatory qPCR results,n=3. *p <0.05, **p < 0.01, ***p <0.001.

A total of 1384875 spectrums were processed using Proteome Discoverer software. On matching 230372 spectrums to Oreochromisniloticus Uniprot database 30736 peptides (28353 unique peptides) were obtained. A total of 4709 proteins were identified and 3345 of them were quantifiable (**Figure 5A**). The Venn diagram showed that there were 100 up-regulated and 84 down-regulated differentially expressed proteins in PCOS *vs.* NC group while 48 upregulated and 78 downregulated differentially expressed proteins were in the NaBu group *vs*. PCOS group. There were 83 overlapping differentially expressed proteins (DEPs), and 30 were upregulated and 53 were downregulated after NaBu treatment (**Figure 5B**). KEGG enrichment analysis was performed on these 83 DEPs using STRING database, and the most significant first 11 gene subsets were obtained, including metabolism of xenobiotics by cytochrome P450, PPAR signaling pathway, cholesterol metabolism, fatty acid degradation, tryptophan metabolism, steroid hormone biosynthesis, complement and coagulation cascades, peroxisome, ferroptosis, lysosome, pentose and glucuronate interconversions. Among them, we focused on partially differentially expressed proteins in the metabolism of xenobiotics by cytochrome P450 and steroid hormone biosynthesis pathways (**Figure 5C**). DEPs are listed in **Supplementary Table S2**. Next, heatmaps were constructed to show the expression patterns of the overlapping DEPs, and found they shared the gene Cyp1b1. Cyp1b1 and Ephx1, which are involved in metabolism of xenobiotics by cytochrome P450, were upregulated in the PCOS rats but recovered in the NaBu rats, whereas Aldh3b1 and Idh1 were downregulated in the PCOS rats and recovered in the NaBu rats. The expression levels of Cytochrome P450 family 11 subfamily a member 1 (Cyp11a1), 3β-hydroxysteroid dehydrogenase (Hsd3b) and Steroidogenic acute regulatory protein (StAR) proteins related to steroid hormone biosynthesis signaling pathway were decreased in the PCOS group, and levels were increased in the NaBu group except Hsd3b; whereas Cytochrome P450 family 17 subfamily a member 1(Cyp17a1) was upregulated in the PCOS rats and reduced in the NaBu rats. We then performed qPCR to validate the DEPs presenting similar expression patterns for the genes selected from proteomic sequencing (**Figures 5D, E**).

### 3.6 The localization and expression of Cyp1b1 in the ovaries of rats and its effects on E2 metabolism

**Figure 6:**
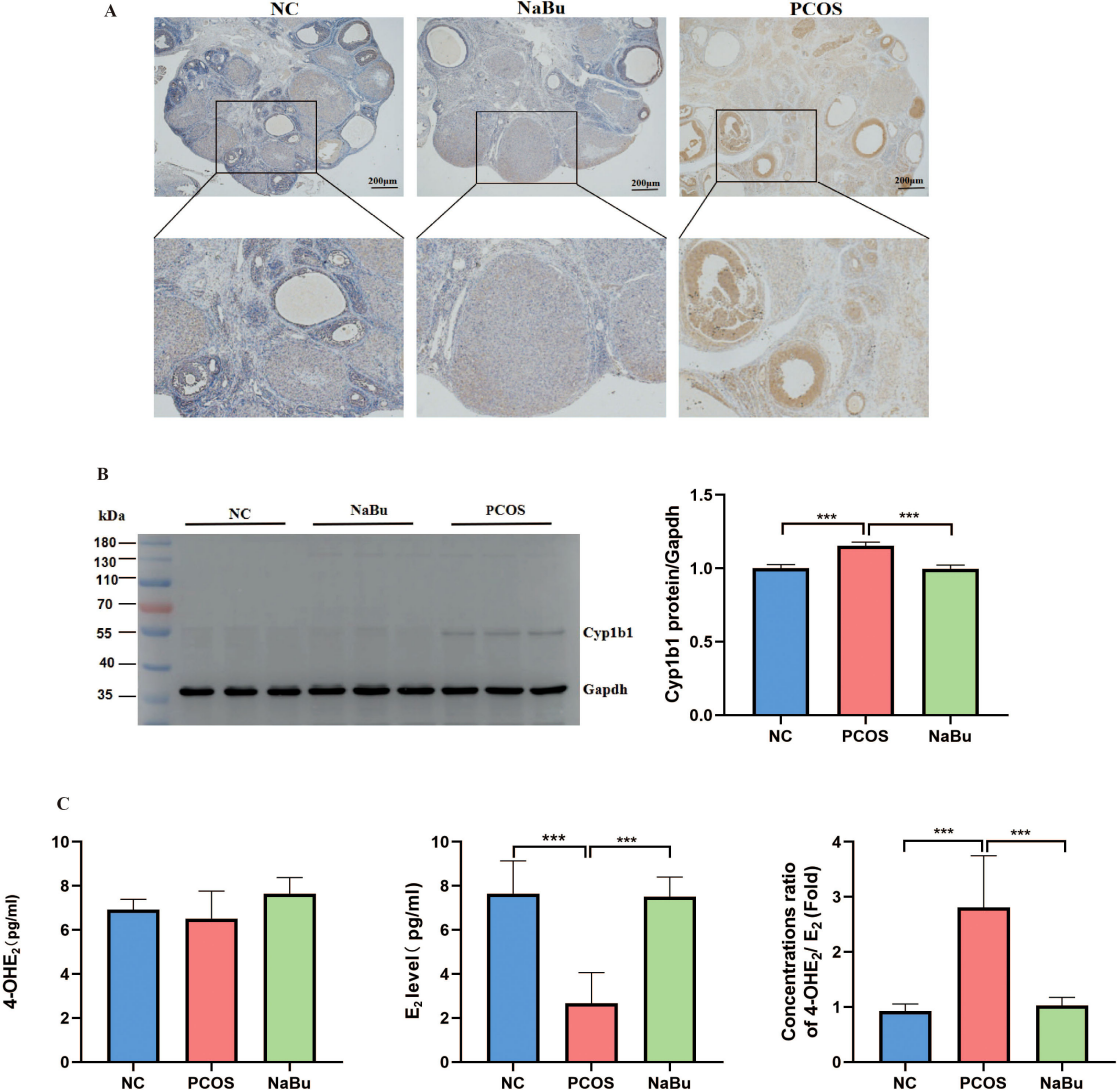
The localization and expression of Cyp1b1 in the ovaries of rats and its effect on E_2_ metabolism. **(A)** Cyp1b1 localization in rat ovary. **(B)** Western blot analysis of Cyp1b1 in rat ovary, n=3. **(C)** The levels of 4-OHE_2_,E_2_ and 4-OH E_2_/E_2_ in rats, n=6. *p <0.05, **p < 0.01, ***p <0.001.

Immunohistochemical and Western blot analyses of rat ovaries revealed that Cyp1b1 expression was lower in both the NC group and the NaBu group, whereas it was significantly upregulated in the PCOS group and predominantly localized in granulosa cells (**Figures 6A, B**). These findings indicate that Cyp1b1 expression is increased in the ovaries of PCOS rats, and its expression is downregulated following NaBu treatment. The serum 4-hydroxy-estradiol (4-OHE_2_) level of rats was detected by ELISA, and the results showed that there was no significant difference in the serum 4-OHE_2_ level of rats in the three groups. However, E_2_ was significantly higher in the NaBu group than in the PCOS group, and therefore the ratio of 4-OHE_2_/E_2_ was significantly higher in the PCOS group (**Figure 6C**).

### 3.7 Overexpression of Cyp1b1 has an inhibitory effect on the function of granulosa cells in rats

**Figure 7:**
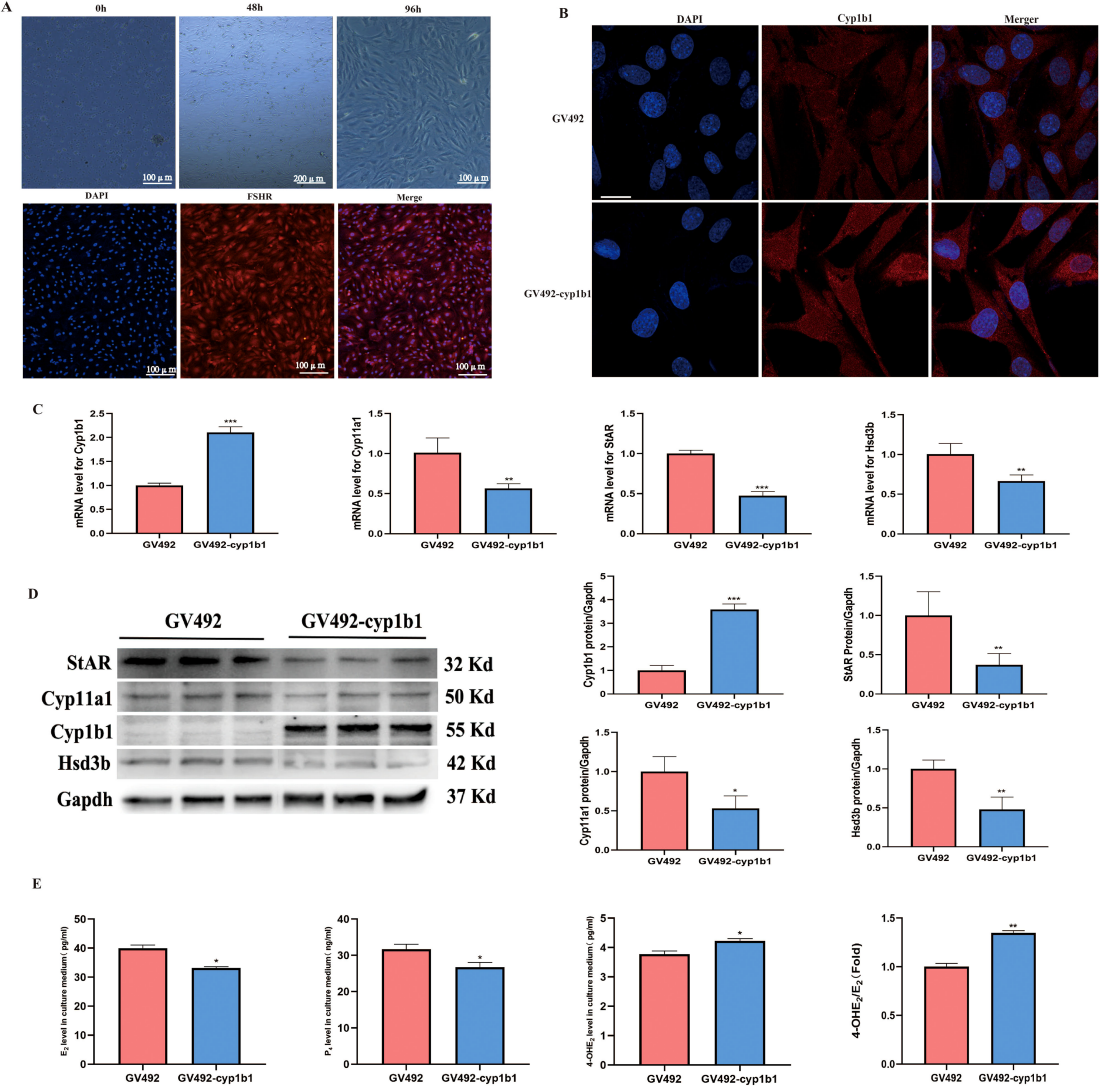
The effects of Cyp1b1 overexpression on rat granulosa cells. **(A)** Culture and identification of rat granulosa cells. **(B)** Verification of infection of lentivirus into rat granulosa cells, scale bar =10 μm. **(C)** RT-qPCR analysis of Cyp1b1, Cyp11a1, StAR, and Hsd3b expression levels in granulosa cells, n=3. **(D)** Western blot analysis of Cyp1b1, Cyp11a1, StAR, and Hsd3b expression levels in granulosa cellss, n=3. **(E)** Levels of E_2_, P_4_, and 4-OHE_2_ were quantified by ELISA, n=3. *p <0.05, **p < 0.01, ***p <0.001.

To investigate the effect of Cyp1b1 overexpression on the mechanism of action in rat ovarian granulosa cells, we conducted primary cell culture. Granulosa cells exhibited excellent growth after being cultured *in vitro* for 96 hours. Immunofluorescence staining revealed that the cell nuclei were stained blue by DAPI, while the FSHR on the cell membrane showed red fluorescence, confirming that the cultured cells were highly pure granulosa cells (**Figure 7A**). Furthermore, we introduced a lentiviral vector into the granulosa cells to achieve overexpression of Cyp1b1. Immunofluorescence results showed that the GV492-cyp1b1 group exhibited stronger fluorescence signals (**Figure 7B**), indicating successful overexpression of Cyp1b1. RT-qPCR analysis revealed that the mRNA levels of Cyp1b1 were significantly higher compared to the GV492 group. Meanwhile, we observed a significant decrease in the mRNA expression levels of Cyp11a1, StAR, and Hsd3b (**Figure 7C**), suggesting that overexpression of Cyp1b1 suppressed the expression of these key steroidogenic enzyme genes. Western blot analysis at the protein level also confirmed this phenomenon: the expression of Cyp1b1 protein was significantly higher, while the protein levels of Cyp11a1, StAR, and Hsd3b were correspondingly lower (**Figure 7D**). To assess the specific impact of these gene expression changes on steroid hormone synthesis, we measured the concentrations of E_2_, P_4_, and 4-OHE_2_ in the granulosa cell culture medium. The results showed that overexpression of Cyp1b1 significantly reduced the secretion of E_2_ and P_4_, while the levels of 4-OHE_2_ and the ratio of 4-OHE_2_ to E_2_ were significantly higher (**Figure 7E**). These data suggest that overexpression of Cyp1b1 not only alters the expression patterns of specific genes in granulosa cells but also affects the biosynthesis pathway of steroid hormones.

### 3.8 NaBu treatment improved reproductive performance of PCOS rats

**Figure 8:**
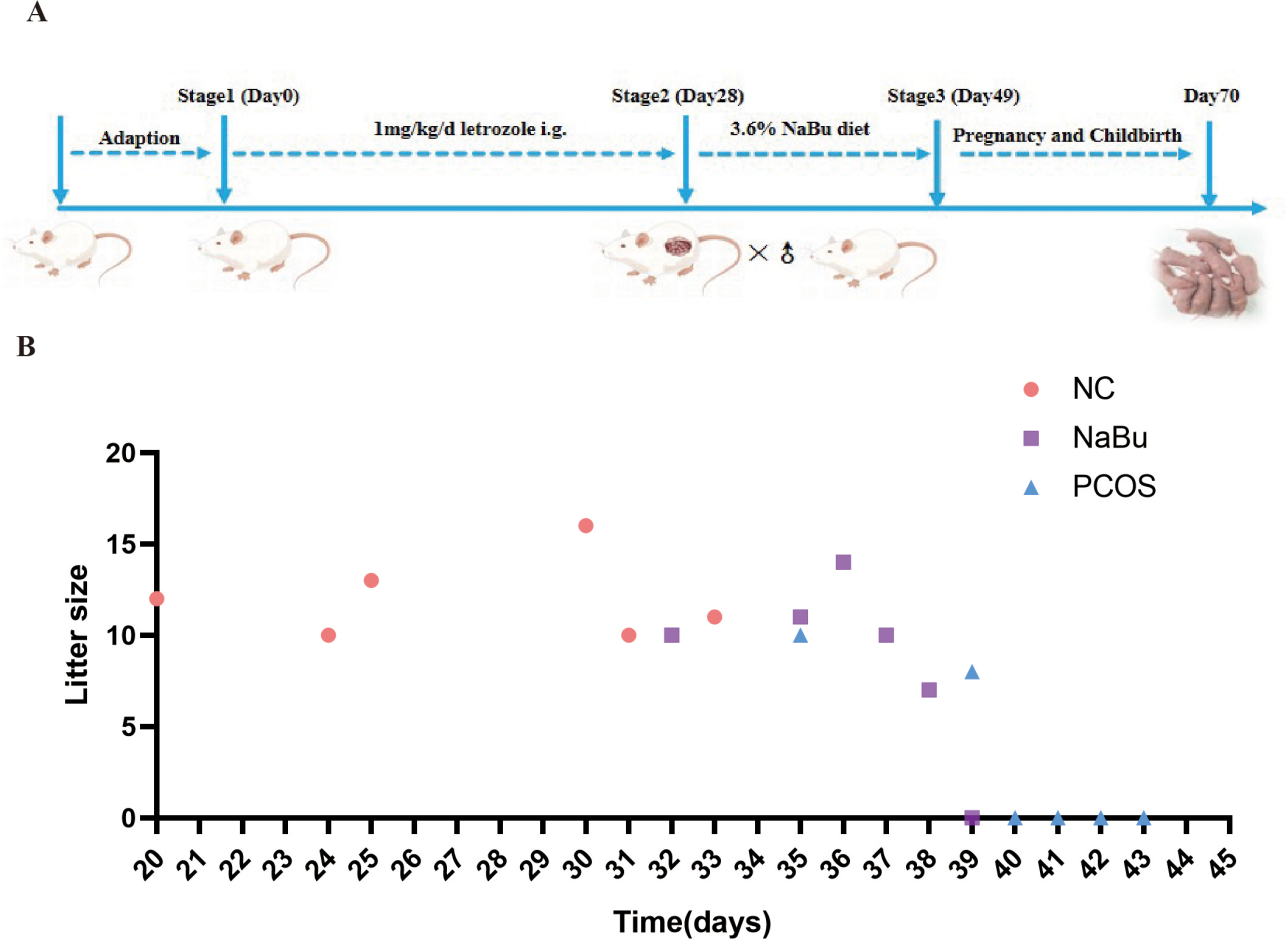
Effects of NaBu treatment on birthing time and litter size in rats. **(A)** Schematic diagram of rat reproduction experiment. **(B)** Breeding records of rats, n=6.

As previously mentioned, dietary addition of NaBu can improve the morphology of PCOS rats’ polycystic ovaries. Here, the beneficial effects of NaBu on ovaries can be further verified by testing the reproductive performance of the rats. Based on the breeding experiment procedure(**Figure 8A**), the rats in the NC group began to give birth on the 20th day after cage confinement, and all 6 rats gave birth on the 33rd day after cage confinement. The rats in NaBu group began to give birth on the 31st day after the cage was closed, and there was still one unpregnant rat within the set time. In the PCOS group, one rat gave birth on day 35 and 39 respectively, and the other four rats were not pregnant (**Figure 8B**). The gestation period of rats generally lasted from 19 to 21 days. In the set time (42d), all the rats in the NC group gave birth, the average litter size was 12, and the reproductive rate was 100%. In the NaBu group, 5 rats gave birth, the reproductive rate was 83.3%, and the average litter size was 10.2. In the PCOS group, only 2 litter were born, the reproduction rate was only 33.3%, and the average litter size was 9 (**Table 1**). These results suggest that NaBu treatment can improve the reproductive performance of PCOS rats.

**Table 1:** Reproductive performance of rats (n=6).

Group	Litter size						Pups	Pups/litters	Reproductive rate(%)
NC	10	12	13	16	10	11	72	12	100%
NaBu	10	11	14	10	7	0	52	10.2	83.30%
PCOS	10	8	0	0	0	0	18	9	33.30%

Reproduction rate = number of litters born/n.

## 4 Discussion

In our study, compared to the NC group, PCOS rats exhibited significant weight gain and abnormal lipid metabolism, as evidenced by elevated levels of TG and LDL-C. These findings are consistent with previous reports (30, 31). Notably, a prior study demonstrated that administration of NaBu significantly improved glucose homeostasis and reduced serum levels of TG, LDL-C, and insulin in female rats following a 6-week high-fat diet (32). In agreement with these results, dietary supplementation with 5% NaBu was shown to effectively lower both TG and TC levels in obese mice. Furthermore, NaBu treatment suppressed weight gain, attenuated the rise in insulin levels, and enhanced insulin sensitivity in these animals (33). Building upon these observations, our findings further confirm that NaBu effectively reduces serum TG and LDL-C levels while inhibiting weight gain in PCOS rats. However, the roles of LDL-C and TG in the pathogenesis of complications in women with PCOS are not yet fully understood (34).

Hyperinsulinemia and insulin resistance are known to promote high androgen levels in PCOS patients (35). The ovarian theca cells produce large amounts of androgens to prevent follicle maturation, which leads to the formation of polycystic ovaries morphology (36). Clinical studies demonstrate that women with PCOS exhibit increased gonadotropin-releasing hormone (GnRH) pulsatility, which drives elevated LH secretion. This heightened LH stimulation subsequently promotes ovarian androgen overproduction. On one hand, LH activates the expression of Cyp17a1 in follicular theca cells by binding to the LH receptor on follicular theca cells, and catalyzes intracellular cholesterol into androgen. On the other hand, LH can induce the ovaries to secrete insulin-like growth factor 1 (IGF-1) through paracrine or autocrine mode, promoting the synthesis and release of androgens. High levels of LH inhibit the function of FSH, lead to premature luteinization of granulosa cells, arrest of the development of small sinusoid follicles, and high levels of androgens, eventually leading to polycystic morphology in the ovary (37). Therefore, the synergistic action of LH and FSH is essential for normal ovarian function, controlling follicle growth, ovulation, and luteum production. Reduced P_4_ levels in women with PCOS are associated with luteal phase insufficiency. In our study, PCOS rats exhibited characteristic polycystic ovarian morphology accompanied by decreased corpus luteum numbers. These pathological changes may be attributed to elevated androgen, insulin and LH levels coupled with reduced E_2_ and P_4_ concentrations. Notably, NaBu intervention effectively reversed these endocrine disturbances, ameliorated polycystic ovarian morphology, and promoted follicular development and ovulation.

Studies have shown that butyric acid induces colonic L cells to express intestinal hormones PYY, GLP-1 and glucose-dependent insulin polypeptide (GIP) through a GPR41-dependent mechanism, which are key regulators of energy homeostasis and glucose metabolism. PYY delays gastric and gallbladder empties, inhibits gastric acid and pancreatic secretion, slows colon transport, and has been shown to suppress appetite (38, 39). PYY also exerts anorexic effects directly on neuropeptide Y2 receptors in the hypothalamic arcuate nucleus (40) or indirectly through neuropeptide Y2 receptors on the vagus nerve (41). In addition, it can bind to the neuropeptide Y5 receptor (Y5R) to inhibit LH secretion in castrated and adolescent rats (38, 42). After intraperitoneal injection of PYY in free-fed rats by Rachel et al., the food intake and weight gain of the rats were significantly reduced; In humans, normal postprandial concentrations of PYY infusion can significantly reduce appetite, reducing food intake by 33% within 24 hours (43). These studies suggest that PYY plays an important role in neuroendocrine regulation and energy metabolism of the reproductive axis. Low levels of PYY were found in serum of PCOS patients, which was negatively correlated with LH and INS (9, 44). Similarly, Lin et al. also found that serum PYY in PCOS patients was negatively correlated with INS, BMI and testosterone (45). Interestingly, in our study, serum PYY levels were lower in PCOS rats, while T, LH, and INS levels were higher. After treatment with NaBu, the disorder of these hormones was restored, which may be caused by the activation of GPR41 by NaBu, promoting the secretion of PYY by colon L cells, thereby inhibiting hypothalamic appetite and gonadal center, reducing feed intake, leading to weight loss, and reducing the stimulation of LH on ovarian theca cells. Weight loss improves metabolic syndrome, androgen excess, and reproductive function (46). Moreover, the study of He et al. also proved that butylated starch can activate GPR41 receptor in the colon of PCOS rats by releasing butyric acid, promote the secretion of PYY, reduce feed intake of PCOS rats, inhibit weight gain and LH secretion, and indirectly improve endocrine disorders and polycystic ovary pathology (31), which is consistent with our research results.

Our proteomic analysis of rat ovaries revealed that Cyp1b1 upregulation significantly disrupts the metabolic and synthetic pathways of E_2_ and P_4_, a finding further validated by subsequent cellular experiments Cyp1b1 catalyzes the E_2_ reaction to produce 4-OHE_2_ and 2-hydroxy-estradiol (2-OHE_2_). As the most active of endogenous estrogen metabolites, 4-OHE_2_ is a carcinogen, it can oxidize catechol estrogens into active semi-quinone and quinone intermediates, and then combine with DNA to form admixtures which causing DNA damage (47). Cyp1b1 is highly expressed in the testis of adult rats, compared with intact rats of the same age, pituitectomy reduced the level of Cyp1b1 protein in the testis of adult rats by 69%. However, subcutaneous injection of LH increased the expression of Cyp1b1 in the testis of pituitectomy rats, but did not recover to the level of intact adult male rats. Treatment of pituitary-excised rats with testosterone propionate caused a small increase in the expression level of the Cyp1b1 protein. In contrast, treatment of intact adult male rats with estradiol benzoate reduced their Cyp1b1 protein expression levels by 91%, suggesting that Cyp1b1 protein expression is regulated by LH and estrogen (48). In addition, Dasmahapatra et al. found that in the ovaries of rats, a surge in LH during pre-estrus resulted in a significant increase in Cyp1b1 mRNA and a significant decrease in Cyp1b1 mRNA during estrus (49). These studies have shown that higher LH levels can promote the expression of Cyp1b1, which is less affected by androgens, while estrogen inhibits its expression. Aldh3b1 is a member of the aldehyde dehydrogenase (ALDH) superfamily that catalyzes the oxidation of aldehydes to carboxylic acids to ensure that toxic aldehydes do not accumulate in the body (50). Studies have shown that Aldh3b1 efficiently metabolizes and protects cells from lipid peroxide-derived aldehydes and oxidants, suggesting that the enzyme plays an important role in the cell’s defense against oxidative stress and downstream aldehydes (51). Compared with empty vector transfected cells, HEK293 cells transfected with Aldh3b1 showed significant protective effect against the cytotoxicity induced by lipoperoxidation product octyl aldehyde (52). Ephx1 exists in the endoplasmic reticulum of cells and has functions of detoxification, catabolism and regulation of signaling molecules (53). Qing et al. found that reduced methylation levels in the Ephx1 promoter region in PCOS patients activated the expression of Ephx1, thereby inhibiting androgen conversion to E_2_ and increasing the risk of PCOS (54). Up-regulation of Ephx1 was also found in the ovaries of obese mice (55). In extra-ovarian tissue, insulin has been shown to activate liver Ephx1 expression (56, 57). Idh1, an important enzyme in the tricarboxylic acid (TCA) cycle, plays a key role in maintaining cellular redox balance by converting isocitrate to α-ketoglutaric acid to produce NADPH. Reduced Idh1 expression disrupts NADPH homeostasis, leading to oxidative stress damage or enhanced cellular sensitivity to oxidative stress (58). Idh1 knockdown suppressed KGN cell proliferation and accelerated senescence, while significantly elevating ROS levels, inducing autophagy activation, and causing cell cycle arrest at S and G2/M phases (59). Furthermore, Idh1 downregulation was linked to follicular atresia (50). Wang et al. reported a significant positive correlation between granulosa cell Idh1 expression and high-quality embryo rates (60).

Clinical studies have shown that increased activity and expression of Cyp17a1 may be one of the causes of hyperandrogenemia in PCOS patients (61). LH stimulates Cyp17a1 mRNA expression and androgen production in ovarian theca cells by activating PI3K/Akt pathway (55). StAR regulates the transport of cholesterol from the outer membrane to the mitochondria (62), where it is converted to pregnenolone by Cyp11a1 (63) and then catalyzed to P_4_ by Hsd3b (64). Cyp17a1 plays a key role in steroid synthesis by converting P_4_ into androgens, which are then further catalyzed by Cyp19a1 into E_2_ (65, 66). E_2_ and P_4_ levels were significantly reduced in PCOS patients, accompanied by decreased StAR expression in human luteinized granulosa cells (67, 68). These findings align with our observations in PCOS rats showing diminished P_4_ levels and downregulated StAR expression. Our *in vitro* experiments further confirmed that the up-regulation of Cyp1b1 expression level inhibited the function of granule cells, such as the decreased levels of E_2_ and P_4_, the decreased expression levels of steroid synthesis-related factors, and the increased levels of 4-OHE_2_ and 4-OHE_2_/E_2_. Therefore, decreasing Cyp1b1 activity may be a therapeutic strategy for ovarian dysfunction.

In mammalian follicles, Cyp1b1 can catalyze the conversion of E_2_ to 4-OHE_2_, and the increase of 4-OHE_2_/E_2_ ratio will inhibit follicle development and lead to atresia. High concentration of 4-OHE_2_ can induce apoptosis and even death of mouse granulosum cells *in vitro*, and down-regulating the expression of Cyp1b1 is the key to maintaining E_2_ levels in mouse dominant follicles (69). 4-OHE_2_ has carcinogenic activity and can induce kidney cancer and uterine adenocarcinoma in rodents (70, 71). Examination of microsomal E_2_ hydroxylation in human breast cancer shows that the ratio of 4-OHE_2_/2-OHE_2_ in tumor tissue is significantly higher than in adjacent breast tissue (72). These studies support the causative role of 4-OHE_2_ in humans and animals, and suggest that Cyp1b1 plays a key role in the causative process. In our study, the serum ratio of 4-OHE_2_/E_2_ in PCOS rats was significantly higher than that in the NaBu group. This observation suggests a potential association between NaBu-mediated Cyp1b1 inhibition and reduced 4-OHE_2_ synthesis. While these data do not establish a causal relationship, the concomitant reduction in Cyp1b1 expression and 4-OHE_2_ levels is consistent with the plausible mechanism whereby NaBu may protect granulosa cell secretory function and maintain normal follicular growth by modulating Cyp1b1 activity. This potential mechanism could contribute to the observed phenotypic differences between groups, wherein NaBu-treated rats exhibited more ovarian corpora lutea and fewer cystic follicles compared to PCOS rats.

Previous studies have found that dietary addition of NaBu can improve the embryo survival rate and fetal number of pregnant rats, enhance the antioxidant capacity of maternal serum, placenta and fetus, promote the synthesis of ovarian progesterone, promote embryo implantation and maintain pregnancy, and reduce early pregnancy loss (29, 73); promote earlier placental discharge and uterine recovery in pregnant cows, shortening the time required for the next estrus and mating (74). Our study also proved that NaBu can improve the reproductive performance of PCOS rats, which is reflected in that the litter birth rate and litter size are significantly higher than PCOS rats, and the reproductive time is earlier than PCOS rats.

**Figure 9:**
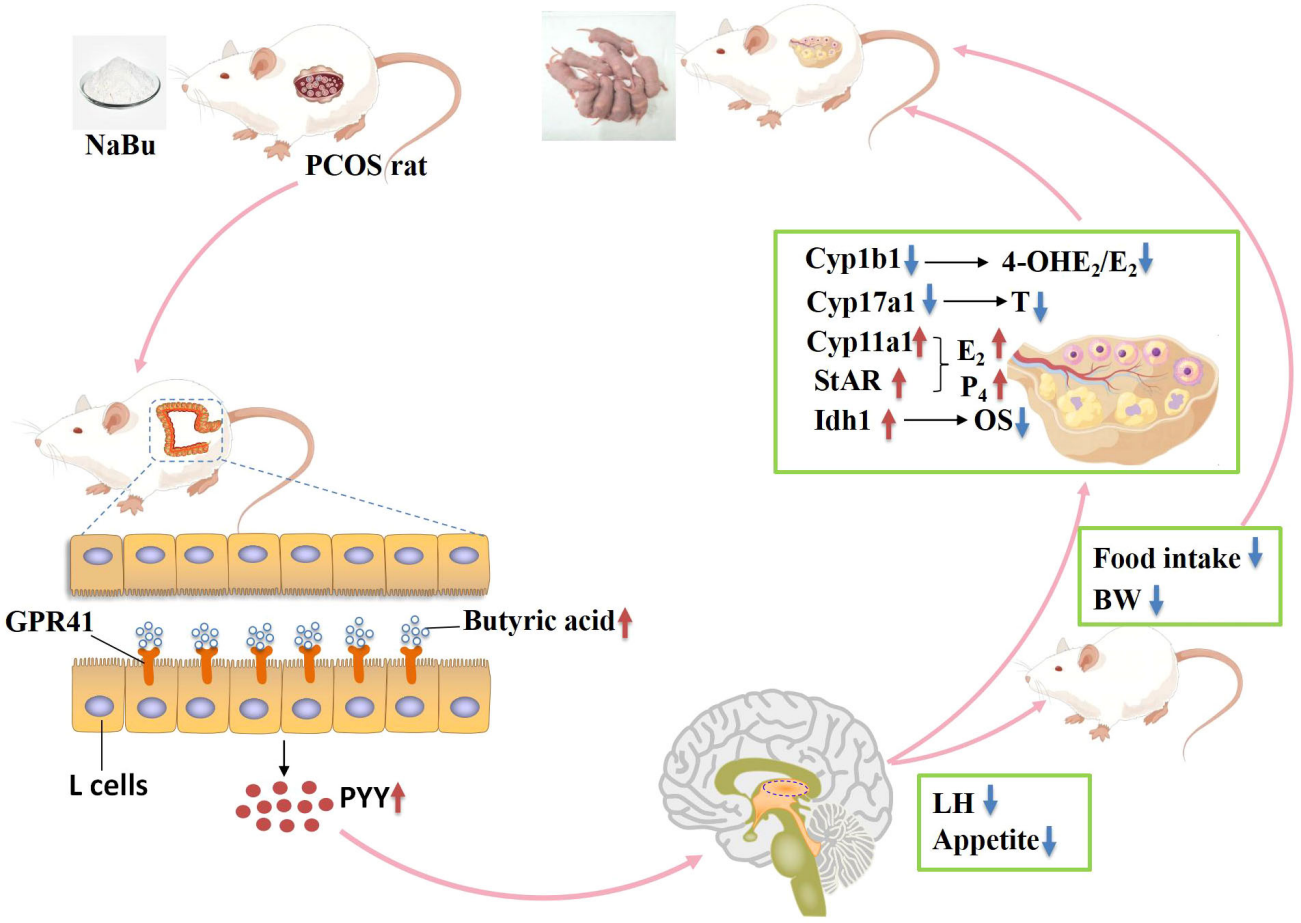
Potential mechanisms of NaBu improving ovarian function in PCOS rats. OS indicates oxidative stress, BW indicates body weight.

To sum up, when lipo-coated NaBu enters the digestive tract of PCOS rats with feed, it can reach the colon of PCOS rats and release, causing an increase in the level of butyric acid in feces. Butyric acid can bind to its specific receptor GPR41, promoting the secretion of PYY by colonic L-cells. PYY, upon binding to its receptor in the hypothalamus through the bloodstream, influences appetite and LH regulation. On one hand, lower appetite inhibits food intake, leading to weight loss and improvement in abnormal lipid metabolism. On the other hand, reduced LH levels weaken stimulation of ovarian theca cells, inhibit Cyp17a1 activity, and decrease androgen synthesis. Concurrently, reduced LH levels downregulate Cyp1b1 expression, which reduces its catalytic activity in converting E_2_ to 4-OHE_2_, thereby lowering the 4-OHE_2_/E_2_ ratio. This protective effect mitigates 4-OHE_2_ toxicity in granulosa cells, while upregulating ovarian expression of Idh1, Cyp11a1, and StAR. Enhanced antioxidant capacity further promotes granulosa cell secretion of E_2_ and P_4_, ultimately ameliorating PCOS phenotypes in rats (**Figure 9**).

## 5 Conclusion

In our study, NaBu may exert its regulatory effects on appetite and hormone levels in the hypothalamus through the gut-brain-ovary axis, modulating the expression of ovarian steroidogenic factors, thereby improving follicular development and granulosa cell function, and enhancing reproductive performance in PCOS rats.

## Data Availability

The datasets presented in this study can be found in online repositories. The names of the repository/repositories and accession number(s) can be found below: https://ngdc.cncb.ac.cn/, OMIX009367.
