# Supplementary material for: Butyric acid ameliorates PCOS-related reproductive dysfunction through gut-brain-ovary axis signaling and ovarian steroidogenic factor activation
Source: Front Endocrinol (Lausanne). 2025 Jul 9;16:1604302. doi: 10.3389/fendo.2025.1604302 (PMC12283308; doi:10.3389/fendo.2025.1604302)
Supplement: Supplementary file 1 [file Table1.doc]

Table S1 The qPCR primer information

| **Primer name** | **Primer sequence（5'-3'）** |
| --- | --- |
| StAR  Aldh3b1  Cyp1b1  Idh1  Ephx1  Cyp11a1  Cyp17a1  β-actin | F: CGTGGCTGCTCAGTATTGACCTC  R: CAAGTGGCTGGCGAACTCTATCTG  F: CTGCGGTCTTGGATGAAGGATGAG R: GAGGCACGAGTGTCAGGTTCAAG  F: GAGAGTTGGTGGCAGTGTTGGTG  R: CTCGGCATCGTCGTGGTTGTACC  F: TTGAAGAAGGTGGTGGTGTTGCC R: AGAGCCATTTGGAAGGAACTGTGTG  F: CATCCAAGCCACCAAGCCAGAC R: TCCATCCTCCAGTTCACGGTACTC  F: GATGCTGCGGGCTGAAGTTCTAG  R: AGTGTCTCCTTGATGCTGGCTTTG  F: ATCCGAGAAGTGCTGCGTATC  R: GGCATGAACTGATCTGGCTG  F: CGATGGGAAGTGCTGGATAG  R: CGGTTAGAGTAGGTGACGTTG |
