# Supplementary material for: Butyric acid ameliorates PCOS-related reproductive dysfunction through gut-brain-ovary axis signaling and ovarian steroidogenic factor activation
Source: Front Endocrinol (Lausanne). 2025 Jul 9;16:1604302. doi: 10.3389/fendo.2025.1604302 (PMC12283308; doi:10.3389/fendo.2025.1604302)
Supplement: Supplementary file 2 [file Table2.doc]

Table S2 Relative quantification of differently expressed proteins in NC, NaBu, and PCOS groups using data-dependent acquisition analysis

| **Protein accession** | **Gene name** | **Protein description** |  | **PCOS/NC**  **Ratio *p-*value** | **NaBu/PCOS**  **Ratio *p-*value** |  |
| --- | --- | --- | --- | --- | --- | --- |
| P07687 | Ephx1 | Epoxide hydrolase 1 |  | 2.063 0.001 | 0.475 0.001 |  |
| A0A0G2K7W2 | Aldh3b1 | Aldehyde dehydrogenase |  | 0.703 0.043 | 1.309 0.017 |  |
| P41562 | Idh1 | Isocitrate dehydrogenase [NADP] cytoplasmic |  | 0.638 0.001 | 1.454 0.005 |  |
| **Q64678** | **Cyp1b1** | **Cytochrome P450 1B1** |  | **2.422 0.015** | **0.369 0.006** |  |
| P22072 | Hsd3b | 3 beta-hydroxysteroid dehydrogenase |  | 0.737 0.044 | - - |  |
| A0A0H2UHG1 | Cyp11a1 | Cholestero iside-chain cleavage enzyme |  | 0.533 0.003 | 1.576 0.001 |  |
| P97826 | Star | Steroidogenic acute regulatory protein |  | 0.526 0.009 | 1.445 0.016 |  |
| A0A0G2JSR8 | Cyp17a1 | Cytochrome P450, family 17, subfamily a, polypeptide 1 |  | 4.725 0.001 | 0.249 0.001 |  |
